# Evaluating Patient Preferences and Clinical Outcomes for Modified Laparoscopic Burch Colposuspension and Transobturator Tape Procedures in Stress Urinary Incontinence Treatment

**DOI:** 10.3390/life14030380

**Published:** 2024-03-14

**Authors:** Simona Brasoveanu, Ligia Balulescu, Dorin Grigoraș, Dragos Erdelean, Flavius Olaru, Răzvan Bardan, Oana Balint, Mădălin-Marius Margan, Alexandru Alexandru, Ivan Cristiana-Smaranda, Laurențiu Pirtea

**Affiliations:** 1 Department of Obstetrics and Gynecology, Victor Babes University of Medicine and Pharmacy, 300041 Timisoara, Romania; simona.brasoveanu@umft.ro (S.B.); grigorasdorin@ymail.com (D.G.); erdelean.dragos@umft.ro (D.E.); olaru.flavius@umft.ro (F.O.); balint.oana@umft.ro (O.B.); pirtea.laurentiu@umft.ro (L.P.); 2 Department of Urology, Victor Babes University of Medicine and Pharmacy, 300041 Timisoara, Romania; bardan.razvan@umft.ro; 3 Department of Functional Sciences, Discipline of Public Health, Victor Babes University of Medicine and Pharmacy, 300041 Timisoara, Romania; margan.madalin@umft.ro; 4 General Medicine, Victor Babes University of Medicine and Pharmacy, 300041 Timisoara, Romania; alexandru.alexandru@student.umft.ro (A.A.); smaranda.ivan@student.umft.ro (I.C.-S.)

**Keywords:** modified laparoscopic Burch, transobturator tape, stress urinary incontinence

## Abstract

Objective: This study aims to provide an in-depth analysis of patient preferences and clinical outcomes associated with two prominent surgical techniques for treating Stress Urinary Incontinence (SUI): the modified laparoscopic Burch colposuspension and the transobturator tape (TOT) procedure. Material and Methods: A prospective cohort study was conducted on 145 patients who recieved surgical treatment for SUI, of which 71 patients (49%) underwent the modified laparoscopic Burch procedure, while 74 patients (51%) received the TOT procedure. Data on clinical characteristics, treatment success rates, and postoperative outcomes were collected and analyzed to understand patient preferences and real-world clinical effectiveness. Results: This study revealed notable differences in patient demographics and clinical characteristics between the two groups. At the 2-year follow-up, a success rate of 100% was reported in the modified laparoscopic Burch group and 86.48% in the TOT group. A total of 99 patients (68.28%) were considered cured postoperatively, with 47 (66.20%) in the modified laparoscopic Burch group and 52 (70.27%) in the TOT group (*p* = 0.598). A significant difference was found in the incidence of dyspareunia, with six cases (8.10%) reported in the TOT group, compared to none reported in the modified laparoscopic Burch group (*p* = 0.028). The median operation time was significantly shorter in the TOT group, namely 15 min, compared to the modified laparoscopic Burch group, which had a median equal to 27 min (*p* < 0.001). Despite these differences, patient preference for either surgical technique was observed, along with similar success rates and varied postoperative outcomes. Conclusions: The findings provide a comprehensive overview of patient preferences and factual clinical outcomes for the two surgical techniques in SUI treatment. This study contributes to understanding the factors influencing patient choice and offers valuable insights into the real-world application of these techniques, enhancing patient-centered care in SUI management.

## 1. Introduction

SUI is defined as involuntary urine leakage during efforts that increase intravesical pressure, such as coughing or sneezing [1]. The prevalence of SUI in women increases with age (14.8–31.8% in women >50 years), with an increasing prevalence during young adult life [1]. The percentage of women who will undergo surgery for urinary incontinence is about 4% [2]. According to estimates, urinary incontinence might possibly impact up to 28.4 million women in the United States by 2050 due to the rise in life expectancy and the significant growth of the adult population in developed countries [3].

For nearly 100 years, surgeons have struggled to find the ideal treatment for this condition and several options have been suggested, including conservative therapy (lifestyle modifications, Kegel exercises, Duloxetine), minimally invasive procedures (sub urethral slings, transurethral bulking agents, radiofrequency collagen remodeling), surgical interventions, and even artificial urinary sphincter placement [4].

There are numerous surgical procedures that aim to elevate and stabilize the urethrovesical junction, creating a hammock-like support beneath the urethra [5].

The primary surgical approach frequently employed for addressing SUI in women is the placement of mesh. This technique saw an increase in mesh units sold globally to approximately 3.7 million units from 2005 to 2013. Within the United States, the Food and Drug Administration (FDA) has put forth a proposal to elevate the risk categorization of urogyneacological meshes, thereby necessitating premarket notification and the implementation of specific controls [6]. Ever since the FDA warnings and the implementation of procedures such as the utilization of peri-urethral bulking agents, meshes have become increasingly popular. The purpose of injecting bulking agents submucosal in the female urethra is to obtain continence by apposition of the urethral wall. Another type of surgery is retropubic suspension procedures like Burch colposuspension. In this type of procedure, the fundamental principle is raising and securing the bladder neck and the proximal urethra in a retrograde position to provide improved support [4] and pubourethral ligaments plication, as presented by Petros P. [7].

In this context, patients are requesting alternative treatments for urinary incontinence as a result of the condemning criticism of synthetic meshes used transvaginal. The Burch colposuspension, invented by John C. Burch in 1961, had been the preferred method for treating female patients with SUI due to its high success rates of 56–88% until the advent of mid-urethral slings in the mid-90s [3,4]. Despite the arrival of new techniques, the Burch colposuspension is experiencing a resurgence in popularity [3,4].

Tension-free vaginal tape (TVT), initially reported in 1995 by Ulmsten U. and Petros P., had been there after the most common surgical treatment for SUI [8].

The TOT surgery, also known as the “outside-inside” technique or as the midurethral sling (MUS) was first described by Delorme in 2001 and had a high rate of success and low perioperative complications, with the aim of preventing serious vascular, bladder, and bowel damage. In particular, it has been reported that the out–in approach is safer for preventing injury to the dorsal nerve of the clitoris [5]. In a decision analysis, Weber and Walters found the overall effectiveness of the Burch procedure and sling procedure to be 94.8% and 95.3%, respectively [9].

The first reported retropubic surgery performed via the laparoscopic approach was described by Vancaillie and Schuessler in 1991 [4]. In 1993, Liu and Paek reported 107 cases of laparoscopic colposuspension with an overall success rate of 97.2% and an overall complication rate of 10.2% and they concluded that the laparoscopic technique is a workable and secure substitute for open surgery [10]. The interest in laparoscopic surgery for retropubic spaces has increased significantly in the past years.

### Objective

The primary objective of this study is to provide a comprehensive overview of patient preferences and clinical outcomes associated with two surgical techniques for treating SUI: the modified laparoscopic Burch colposuspension and the TOT procedure. This study aims to document factual observations from clinical practices, including patient demographics, clinical characteristics, treatment success rates, and postoperative outcomes. By focusing on patient preferences and real-world clinical data, the study seeks to offer insights into the selection of surgical techniques for SUI based on patient-centric and clinical practice perspectives, rather than conducting a comparative efficacy analysis of the two methods.

## 2. Materials and Methods

### 2.1. Patients

We performed a prospective cohort evaluation including all eligible patients who underwent surgery for SUI in the Department of Obstetrics and Gynecology of Timișoara University City Hospital, between January 2019 and December 2020. The patients included in our study underwent either modified laparoscopic Burch colposuspension or the TOT procedure.

### 2.2. Ethical Aspects

This study was conducted after receiving approval from the Human Ethical Committee of the University of Medicine and Pharmacy “Victor Babes”, Timisoara, Romania (Approval Number: 58/12 December 2018), in strict adherence to ethical standards. All interventions carried out in this study involving human participants conformed to the principles set forth in the Declaration of Helsinki (revised in 2013). Written informed consent was obtained from each patient prior to their participation in the study.

### 2.3. Study Design

The choice of patients for either the modified laparoscopic Burch group or the TOT group was based on comprehensive discussions between the patients and their doctors. During these discussions, the potential risks, complications and cure rate from the literature of each procedure were thoroughly discussed, allowing for a more informed and collaborative decision-making process.

The following parameters were evaluated for each patient: body mass index (BMI), parity, post-menopausal status, duration of surgery, postoperative complications, blood loss, hospital stay.

Women with SUI can also experience voiding dysfunctions, such as overactive bladder, dysfunctional voiding, detrusor underactivity, or increased post-void residual volume. These conditions may affect treatment outcomes, resulting in the following inclusion criteria: female patients aged over 18, diagnosed with genuine, symptomatic SUI, normal urethral closing pressure, and positive cough test.

**Figure 1:**
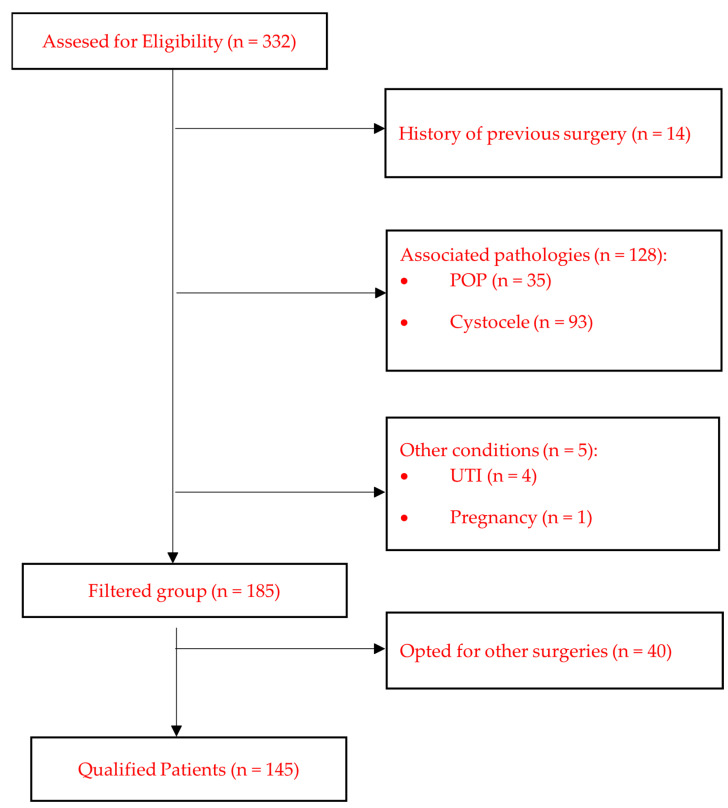
The algorithm for patient recruitment.

Exclusion criteria were female patients with urinary incontinence that had significant urgency and urge urinary incontinence diagnosed using bladder diary, SUI due to low urethral closing pressure. Furthermore, we excluded patients with comorbidities that could bias the results, such as pelvic organ prolapse (POP) greater than grade 1, cystocele, urinary tract infection not responding to treatment, patients with antipsychotic treatment (because urinary retention can develop during antipsychotic treatment), ongoing pregnancy. Patients with previous vaginal repair or recurrent incontinence were also excluded from our study. The algorithm for patient recruitment is presented in Figure 1.

### 2.4. Outcomes of the Study

The primary outcomes of this study were centered around understanding patient preferences and evaluating the success rates of two surgical techniques for managing stress urinary incontinence (SUI). Additionally, an analysis of patient demographics and clinical characteristics was conducted to explore their potential influence on the selection of the surgical method. The success rate was defined by the absence of leakage during the pad test and/or by a negative stress maneuver (cough test or Valsalva test). We used the pad test if the cough test was negative but patient reported leakage during daily activities.

Secondary outcomes included the operation time and hospitalization duration, as well as assessment of intraoperative and short-term complications (bowel perforation, de novo urge incontinence, acute urinary retention) and long-term complications (post void residual volume, dyspareunia, chronic pelvic pain, and mesh erosion).

### 2.5. Surgical Technique

Both procedures were performed by the same surgical team. A single dose of antibiotic for prophylaxis was administrated preoperatively. The Foley catheter was removed 12–24 h after surgery in the TOT group and 24 h after in the modified laparoscopic Burch group.

As follows is a technique description of the personally modified laparoscopic Burch procedure: The standard laparoscopic instruments used are: bipolar forceps, monopolar hook, scissors, atraumatic forceps, and 2 needle holders. We use 2/0 monofilament non absorbable thread with a 3/8 26 mm round needle for the Cooper ligament and 2/0 Vicryl suture for the parietal peritoneum.

**Figure 2:**
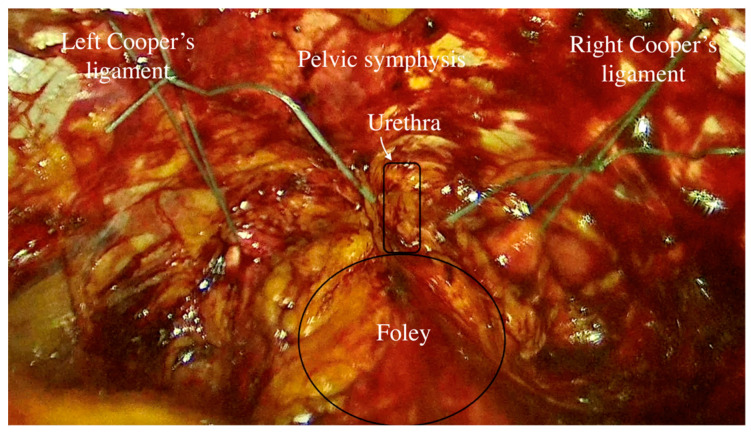
The anatomical landmarks of Retzius space.

**Figure 3:**
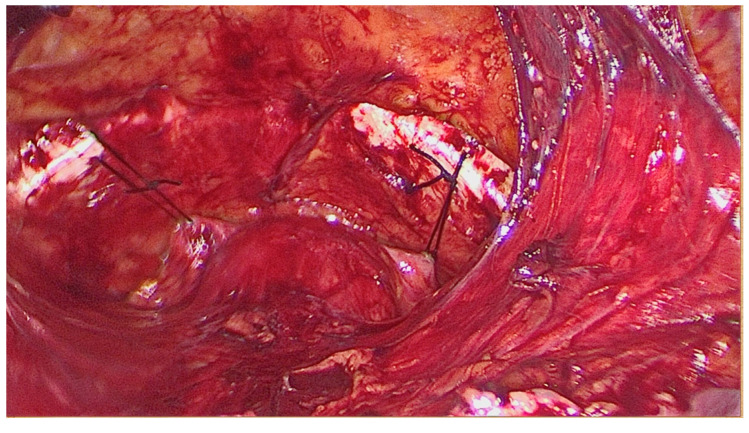
The final aspect of modified laparoscopic Burch colposuspension technique with one thread on each Cooper ligament.

Direct entry is used to induce pneumoperitoneum. Prior to peritoneal incision, the bladder is filled with 250–300 mL saline solution in order to correctly expose the superior limit of the bladder dome to avoid blader injury at this level. Transperitoneal approach of Retzius space is achieved by performing an incision of approximately 5–6 cm of the parietal peritoneum between the two obliterated ombilico-vesical arteries superior to the bladder dome (upper vesical bladder limit). The bladder is drained after the peritoneum is incised and the avascular space of Retzius is developed by blunt dissection. The anatomical landmarks are represented by the pubic symphysis and the Coopers ligaments (Figure 2). The Foley catheter is also taken into account to identify the bladder neck. The vaginal walls are identified by blunt dissection with the aid of the assistant, who is lifting the vaginal wall through the vaginal route. Only one thread is placed on each side as follows: one bite through the medial part of each Cooper ligament and two bites on the vaginal wall caudally to the Foley catheter (in order to be placed in the mid urethral area) (Figure 3). The threads are knotted without creating extensive tension using intracorporeal knots. The parietal peritoneum is closed using a continuous suture. No drainage is needed. The Foley catheter is removed 24 h after surgery.

The standard technique for TOT procedure is described by Delorme [11]. Technique description: The TOT standard procedure is performed under spinal anesthesia. The patient is placed in lithotomy position with hyper flexion of the hip. A 2 cm incision is made on the anterior vaginal wall over the miduretha. The blunt dissection continues laterally until the index finger comes in contact with the internal surface of the ischiopubic bone and obturator foramen. A horizontal line is drawn from the level of clitoris to the inguinofemoral sulcus on both sides. A 1 cm vertical skin incision is made where the line crosses the sulcus. Using specially designed needles, the obturator membrane is perforated and then the needle is turned medially. It is then guided with a finger in the vaginal incision to exit in the vagina. The tape is then loaded onto the needle and pulled through the skin incision on each side. Tension is adjusted so that a dissecting scissors can lie flat easily between the tape and the urethra. The incisions are closed with 2.0 Vicryl. The Foley catheter is removed 12–24 h after surgery.

### 2.6. Follow-Up

The follow-up period comprised evaluation at 1, 12, and 24 months after the procedure. The follow-up visits targeted the following parameters: success rate, complications: post void residual volume, dyspareunia, mesh erosion, and chronic pelvic pain.

### 2.7. Statistical Analysis

For medical data collection, management, and analysis, we employed the Mediflux™ software v1.1. (developed by Origini Health™, Amsterdam, The Netherlands). Statistical computing was performed using Python v3.9.13 (Python Software Foundation™). The Pandas library was used for data manipulation, and the SciPy library was used for statistical analysis.

Descriptive statistics: Continuous variables were summarized as means with standard deviation or medians and interquartile range (IQR), depending on which was more appropriate, while categorical variables were presented as counts and percentages. Normality Assessment: Continuous variables distribution was assessed using the Shapiro–Wilk test.

Group Comparisons: Mann–Whitney U test *p*-value was applied to compare the means of continuous and ordinal variables across the two patient groups. The Mann–Whitney U test is a non-parametric statistical test, which means it does not assume a normal distribution of the data. This test is particularly adept at comparing the medians of two independent samples. For categorical variables, due to the small sample sizes in the groups, Fisher’s Exact Test was predominantly utilized. The Chi-square test was employed in instances where the expected frequencies in each cell of the contingency table surpassed five. A *p*-value of less than 0.05 was considered statistically significant.

To explore the influence of clinical factors on surgical preferences, we employed propensity score calculation for each patient and visually assessed propensity score distributions against a considered factor using scatter plots. A multivariate logistic regression model tried to ascertain potential risk factors for operative failure.

Methods Limitations:

The limited sample size in our study, a result of practical constraints, may indeed impact the statistical power, potentially making it more challenging to detect subtle differences between groups. This aspect could limit the generalizability of our findings and suggests caution in interpreting the results, particularly when extrapolating to a broader population. Nonetheless, the detailed evaluation of each participant ensures robustness in our findings, adding a layer of reliability to the data within the confines of the study’s scope. The decision to summarize continuous variables with means and standard deviations or medians and IQR depending on normality can introduce inconsistency. Comparing means assumes a normal distribution, while medians are more appropriate for skewed data. This duality can complicate the interpretation of results.

The findings from the study may not be generalizable to all patient populations, especially if the study population has unique characteristics or if the sample size is not representative of the broader population of patients with SUI.

## 3. Results

Primary Outcomes:

Patient Preferences: The study analyzed the preferences of 145 patients, with 71 opting for the modified laparoscopic Burch technique and 74 for the TOT intervention. The choice was made after the patients’ discussions with their doctors, ensuring the patients understand the potential risks and cure rates of each surgery type.

The demographic statistics and clinical characteristics of the 145 patients included in the study are presented in Table 1. Significant differences were observed for age, BMI, and parity between the two groups. The mean age of patients in the modified laparoscopic Burch group was 48.02 years ± 4.71 years, significantly lower than the mean age of 66.04 ± 6.55 years in the TOT group.

Furthermore, patients in the TOT group had a higher mean parity of 2.27 ± 0.89, compared to a mean parity of 1.67 ± 0.65 in the modified laparoscopic Burch group (*p* < 0.001). Regarding menopausal status, there was a significantly higher proportion of postmenopausal women in the TOT group (91.89%) compared to the modified laparoscopic Burch group (28.17%) (*p* < 0.001).

**Figure 4:**
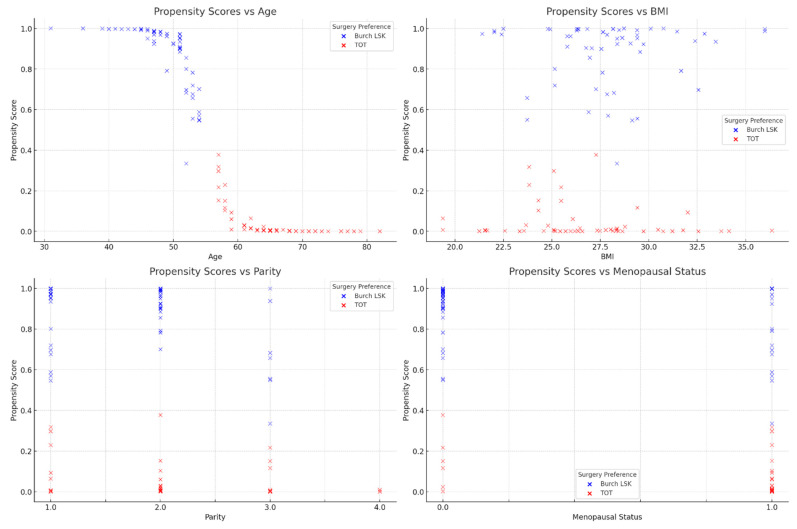
Relationship between propensity scores and clinical factors by surgery type preference.

In addition, the propensity score scatter plots (presented in Figure 4) do depict a clear-cut pattern for age and obvious trends for parity and menopausal status, emphasizing the nature of patient decision-making in the context of SUI surgery.

Success Rate:

In terms of cure rate, there was no significant difference between the two groups, as shown in Table 2. A total of 99 patients (68.28%) were considered cured postoperatively, with 47 (66.20%) in the modified laparoscopic Burch group and 52 (70.27%) in the TOT group (*p* = 0.598). Significant clinical improvement was observed in 34 patients (23.45%), 18 (25.35%) in the modified laparoscopic Burch group and 16 (21.62%) in the TOT group (*p* = 0.596). Treatment failure was reported in 12 patients (8.28%), with an equal distribution in both groups (*p* = 0.921).

Follow-up visits at one month were completed by all patients (100%) in both groups.

At the one-year follow-up visit, 141 patients (97.24%) returned, including all 71 patients in the modified laparoscopic Burch group and 70 patients (98.59%) from the TOT group. At the two-year follow-up visit, 135 patients (93.10%) participated, including all in the modified laparoscopic Burch group and 64 (86.48%) in the TOT group, a statistically significant difference.

In the multivariate logistic regression model employed to identify potential risk factors for operative failure, which includes surgery type (Burch vs. TOT) as well as the other covariates: age, menopausal status, parity, and BMI, all of these factors failed to exhibit a statistically significant influence. The analysis suggests that, after controlling for the included covariates, there is no strong evidence to favor one surgical method over the other in terms of the likelihood of being cured.

Secondary Outcomes:

Peri-operative outcomes (presented in Table 3) showed no significant difference in hemoglobin loss between the modified laparoscopic Burch group and TOT group, with a median loss of 0.9 g/dL (IQR = 0.69–1.04) and 0.86 g/dl (IQR = 0.66–1.19), respectively, (*p* = 0.868).

Significant differences were observed regarding operation time and hospitalization duration. The median operation time was significantly shorter in the TOT group, with a 15 min median (IQR = 12.25–18.00) compared to the modified laparoscopic Burch group, with a 27 min median (IQR = 25.00–29.50) (*p* < 0.001). Furthermore, patients in the TOT group had a shorter median hospital duration of just 1 day (IQR = 1–1) compared to the 2 days median (IQR = 2–2) of the modified laparoscopic Burch group (*p* < 0.001).

In terms of intraoperative complications, bowel perforation was reported in one patient (1.40%) from the modified laparoscopic Burch group at the moment of direct entry. The patient had a history of previous surgery with extended adhesions. None from the TOT group (*p* = 0.489) had intraoperative complications (Table 4).

Short post operative complications are the following: de novo urge incontinence and acute urinary retention.

In terms of short-term complications, de novo urge incontinence occurred in two (2.81%) and three (4.05%) patients from the modified laparoscopic Burch group and TOT group, respectively, with no significant difference (*p* = 1.000). Acute urinary retention was reported in one patient from the TOT group only (*p* = 1.000). (Table 4)

Reported long-term complications are post void residual volume, chronic pelvic pain, dyspareunia, and mesh erosion.

In terms of long-term complications, there were four cases (5.63%) of post void residual volume in the modified laparoscopic Burch group at 1 month follow-up and two cases (2.70%) in the TOT group at 1 month follow-up. However, a significant difference was found in the incidence of dyspareunia, with six cases (8.10%) reported in the TOT group, with four cases (5.71%) reported at 12 months follow-up, compared to none in the modified laparoscopic Burch group. Mesh erosion was recorded in four cases (5.40%) in the TOT group, with three cases (4.68%) reported at 24 months follow-up. Chronic pelvic pain was reported with three cases in TOT group, two cases (2.85%) at 12 months follow-up and one case (1.56%) reported at 24 months follow-up. In the modified laparoscopic Burch group, chronic pelvic pain was reported in one case (1.40%) at 12 months follow-up (Table 5).

## 4. Discussion

The Burch procedure was the most used technique since 1961, when it was initially reported, until 1995, when the sub urethral sling was introduced by Petros P. and Ulmsten U. [12,13].

It was designed as open surgery, but several studies demonstrated that it can be performed by laparoscopy, minimizing the trauma related to the procedure. The procedure, as originally described, consisted of placing 2–4 sutures lateral to the urethra and bladder neck on both sides. This can induce more than just a limitation of urethral hypermobility, but also a modification of the angle between urethra and bladder neck. This can generate de novo urgency and incomplete bladder voiding [12].

The modified Burch technique we performed consisted of the placement of a single suture on each side caudally to the Foley catheter. In this way, the suture provides a limitation of hypermobility in the middle third urethra without modifying the angle between the urethra and the bladder neck, generating a plus of resistance in the same area as the sub urethral sling and creating the same effect without the use of the polypropylene material. Compared to the traditional Burch technique, the modified version offers a restoration of anatomy according to the principles of the Integral Theory issued by Petros [14]. This is in concordance with the low rates of de novo urgency and the low rates of voiding difficulties that we found in the patients with the modified Burch technique.

The sub urethral polypropylene sling presented certain advantages, namely a short learning curve, minimally invasive technique, and high cure rates. The use of sub urethral sling generates a limitation of the hypermobility in the middle third of the urethra and, from this point of view, represents a more anatomic approach to the treatment of SUI [13]. However, polypropylene implant-related complications, such as erosion, chronic pain, and dyspareunia should be considered after sub urethral sling procedures [15]. The above-mentioned complications can be associated with errors of surgical technique, or with the lack of experience of the surgeon. However, complications such as Shoenfeld syndrome, an autoinflammatory/autoimmunity syndrome consisting of fatigue, fever, nausea, and chronic pain, can be related to the insertion of the polypropylene material, irrespective of technique or surgeon’s experience [16].

The selection of patients for either the modified laparoscopic Burch group or the TOT group was predicated on thorough consultations between patients and the surgical team. Within these consultations, the prospective risks, complications, and cure rates associated with each procedure were deliberated based on the existing medical literature.

The patient characteristics in this study seem to have played a significant role in influencing their choice of surgery between the modified laparoscopic Burch technique and the TOT intervention. The study’s results show notable differences in age, BMI, parity (number of births), and menopausal status between the two groups, which could have impacted patient preferences.

The mean age in the Burch group was significantly lower (48.02 years) compared to the TOT group (66.04 years). Younger patients might have preferred the Burch technique due to various reasons such as the perception of a quicker recovery, less invasive nature, being afraid of dyspareunia, and mesh erosion. On the other hand, older patients might have opted for TOT due to different considerations such as the procedure’s suitability for older age, their medical history, or the effectiveness of the procedure.

The BMI was slightly higher in the Burch group (27.91) compared to the TOT group (26.85). Although the difference is not drastic, it is possible that patients with a slightly higher BMI might have been guided towards the Burch technique due to specific surgical considerations or expected outcomes related to their weight.

The mean number of births was higher in the TOT group (2.27) compared to the Burch group (1.67). This difference suggests that women who have had more children might be more inclined to choose TOT, possibly due to the nature of their pelvic floor issues, which could be more pronounced after multiple childbirths, making TOT a more suitable option.

There was a significantly higher proportion of postmenopausal women in the TOT group (91.89%) compared to the Burch group (28.17%). Postmenopausal women might have conditions more amenable to the TOT procedure, possibly due to changes in pelvic floor strength and structure with age and hormonal changes.

In terms of cure rate, in our study there was no significant difference between the two groups. A total of 99 patients (68.28%) were considered cured postoperatively, with 52 patients (70.27%) in the TOT group. In their study, Bandarian et al. reported a higher complete cure rate in TOT group with 90.3% [17] and in a prospective trial by Sivaslioglu et al. [5], the cure rates of SUI at one year was 85.7% in the TOT group and the cure rate at the two-year follow-up was similar, 87.5% in the TOT group (32 patients of 49 were available). Asicioglu et al. reported an objective cure rate of 77.5% and subjective cure rate of 81.7% in their TOT group (272 patients) with a 5-year follow-up [8].

Regarding cure rate after the laparoscopic Burch procedure, our study reported a cure rate of 66.2% in the modified laparoscopic Burch group. In their research, Conrad et al. reported higher subjective success rates, with 78.1% of patients reporting no symptoms of SUI at a mean follow-up of 50.6 months and 12.4% reporting significantly improved symptoms in the laparoscopic Burch group [18]. Yang et al. described their high results with a laparoscopic Burch group with 116 of 155 women available at 1-year follow-up with an objective cure rate 94.8% (110/116) and a subjective cure rate 95.7% (110/116) [19], and Hong et al. reported a 72.1% cure rate in their laparoscopic Burch group [20].

The high cure rate can be explained by the short follow-up period.

To our knowledge, the only study that compared the laparoscopic Burch procedure with the TOT procedure was reported by Samiee et al. The study reported an objective cure rate of 75% for the laparoscopic Burch procedure and an objective cure rate of 84% for TOT, measured by the absence of urine leakage during stress tests and confirmed by urodynamic testing [21]. However, the full text of the publication is not available. No other studies comparing these two techniques have been reported so far. Several randomized trials and numerous cohort studies [22,23,24,25] have compared the effects of standard Burch colposuspension and TVT in the surgical treatment of SUI. Also, only two randomized controlled trials [5,17] have compared the efficacy of TOT and the standard Burch colposuspension. The limitation of these studies was that their postoperative follow-up period did not exceed 24–28 months.

In our study, at the two-year follow-up visit, 93.10% participated, including all in the modified laparoscopic Burch group and 86.48% in the TOT group. The lower rate at of two-year follow-up in the TOT group can be explained by the older age of the patients.

The insertion of a polypropylene sling, although very efficient in terms of cure rates for SUI, has reports of long-term complications such as mesh exposure, chronic pain, and dyspareunia, with a negative impact on life quality, especially for sexually active patients [26].

The variations in complications might be influenced by age and menopausal status. For instance, post-menopausal women, more prevalent in the TOT group, could be at a higher risk for certain complications due to factors like decreased estrogen levels, which can affect urethral and bladder function. Additionally, older age might also contribute to variations in surgical outcomes. Further studies are recommended to conclusively determine the causative relationship between age, menopausal status, and complication rates, ensuring that surgical procedures are tailored to individual patient profiles for optimal outcomes.

In terms of long-term complications, our study reported no significant differences between the two groups in post void residual volume and chronic pelvic pain. However, a significant difference was found in the incidence of dyspareunia, with six cases (8.10%) reported in the TOT group compared to none in the modified laparoscopic Burch group. Mesh erosion was recorded in four cases in the TOT group. Multivariate analysis demonstrated that older age, diabetes mellitus, current smoking, length of vaginal incision > 2 cm, recurrent vaginal incision for postoperative complications, and previous pelvic organ prolapse or incontinence surgery were independent risk factors for mesh erosion [27].

In the study of Lukban, 6% (N = 33) of the patients concluded that they were less able to have a sexual relationship after TOT procedure. Also, 14.9% of the patients experienced vaginal pain, pressure, or protrusion [28]. In a 27 months follow-up of a cohort of 233 women who underwent TOT, de novo dyspareunia was reported in 9% of the patients [29].

All studies have reported shorter operation times and hospital duration for the sling procedure compared with laparoscopic Burch colposuspension. The duration of the surgery was significantly shorter, 15.08 (±3.37) minutes, with hospital duration also shorter in the TOT group.

Sivaslioglu et al. and Bandarian et al. reported similar results with operation time for the TOT procedure of 20 (15–25) min and the mean hospital stay in the TOT group was 2.06 ± 1.03 days [5,17]. Ulmsten U. et Petros P. reported similar results in their study [13].

Paraiso et al. reported a significantly greater operating time (defined as incision to final suture) between 107 and 156 min and a hospital stay between 24 and 42 h for the laparoscopic Burch procedure in their study [30], but our study reported a much shorter operative time for modified laparoscopic Burch colposuspension of 27.50 min (±7.99). This shorter operative time can be explained by the laparoscopic skills of the surgeons.

The modified laparoscopic Burch technique offers similar cure rates for SUI and with lower rates of long-term complications. On the other hand, the technique demands skills in laparoscopic suturing and dissection. The long-term cure rates after this technique need larger studies including more patients for proper assessment and a long follow-up period.

The main limitations of the study are its small study population and the short follow-up period. Also, the inclusion of both pre and postmenopausal patients and inclusion of patients who will go for follow up. Those who follow up could present more complications that could bias the results.

Another limitation of our study that is worth mentioning is the fact that it was a single-center study, and the procedures were performed by one surgical team, thereby potentially limiting the generalizability of the findings.

The main strength of our study is that, to our knowledge, there are no previous studies that have provided a comprehensive overview of patient preferences and factual clinical outcomes for the two surgical techniques in SUI treatment.

## 5. Conclusions

The findings provide a comprehensive overview of patient preferences and factual clinical outcomes for the two surgical techniques in SUI treatment. This study contributes to understanding the factors influencing patient choice and offers valuable insights into the real-world application of these techniques, enhancing patient-centered care in SUI management. This represents a topic of interest, given mesh implants are restricted in certain countries and alternatives to mesh use are continuously investigated.

The complication rates for the modified laparoscopic Burch procedure in our study were lower than for the TOT procedure in the studied period. However, the relatively short follow-up period limits the evaluation of long-term comparative outcomes.

## Figures and Tables

**Table 1:** Demographic statistics and clinical characteristics.

Parameter	Mean (SD)/Median (IQR) or N (%)			
	Study Group N = 145	Burch Group N_1_ = 71 (48.97%)	TOT Group N_2_ = 74 (51.03%)	*p*-Value
Age (years)	57.22 (±10.68) 57 (49–65)	48.02 (±4.71) 49 (46–51)	66.04 (±6.55) 65 (61.25–71)	<0.001 ^1,^*
BMI (kg/m^2^)	27.37 (±3.33) 27.55 (25.18–29.15)	27.91 (±3.05) 27.85 (26.29–29.4)	26.85 (±3.52) 26.41(24.88–28.59)	<0.001 ^1,^*
Parity (n)	1.97 (±0.83) 2 (1–3)	1.67 (±0.65) 2 (1–2)	2.27 (±0.89) 2 (2–3)	<0.001 ^1,^*
Post menopause	88 (68.69%)	20 (28.17%)	68 (91.89%)	<0.001 ^2,^*

^1^ Mann–Whitney U test *p*-value; ^2^ Fisher’s exact test *p*-value; * statistically significant. BMI = body mass index; n = number of births; N = number of patients; N_1_ = number of patients for Burch group; N_2_ = number of patients for TOT group.

**Table 2:** Treatment success rate.

Outcome	N (%)			
	Study Group N = 145	Burch Group N_1_ = 71 (48.97%)	TOT Group N_2_ = 74 (51.03%)	*p*-Value
Cure	99 (68.28%)	47 (66.20%)	52 (70.27%)	0.598 ^1^
Significant improvement	34 (23.45%)	18 (25.35%)	16 (21.62%)	0.596 ^1^
Failure	12 (8.28%)	6 (8.45%)	6 (8.11%)	0.921 ^1^
1 Month follow-up Visit	145 (100%)	71 (100%)	74 (100%)	-
1 Year follow-up Visit	141 (97.24%)	71 (100%)	70 (98.59%)	-
2 Year follow-up Visit	135 (93.10%)	71 (100%)	64 (86.48%)	0.009 ^1,^*

^1^ Chi-square U test *p*-value, * statistically significant. N_1_ = number of patients for Burch group; N_2_ = number of patients for TOT group.

**Table 3:** Perioperative outcomes.

Parameter	Median (IQR) or N (%)			
	Study Group N = 145	Burch Group N_1_ = 71 (48.97%)	TOT Group N_2_ = 74 (51.03%)	*p*-Value
Hemoglobin loss (g/dL)	0.87 (0.67–1.08)	0.9 (0.69–1.04)	0.86 (0.66–1.19)	0.868 ^1^
Operation time (min)	20 (15–26)	27 (25.00–29.50)	15 (12.25–18.00)	<0.001 ^1,^*
Hospitalization (days)	2 (1–2)	2 (2–2)	1 (1–1)	<0.001 ^1,^*

^1^ Mann–Whitney U test *p*-value; * statistically significant; min = minute; N = total number of patients; N_1_ = number of patients for Burch group; N_2_ = number of patients for TOT group.

**Table 4:** Intraoperative and short-term complications.

Parameter	N (%)			
	Study Group N = 145	Burch Group N_1_ = 71 (48.97%)	TOT Group N_2_ = 74 (51.03%)	*p*-Value
Bowel Perforation	1 (0.68%)	1(1.40%)	0 (0.00%)	0.489 ^1^
De novo urge Incontinence	5 (3.44%)	2 (2.81%)	3 (4.05%)	1.000 ^1^
Acute Urinary Retention	1 (0.68%)	0 (0.00%)	1 (1.35%)	-

^1^ Fisher’s exact test *p*-value; N = total number of patients; N_1_ = number of patients for Burch group; N_2_ = number of patients for TOT group.

**Table 5:** Long-term complications.

Parameter	Study Group N = 145 Total		Burch Group N_1_ = 71 (48.97%)				TOT Group N_2_ = 74 (51.03%)		
		At 1 Month FU N_1_ = 71	At 12 Months FU N_1_ = 71	At 24 Months FU N_1_ = 71	Total	At 1 Month FU N_2_ = 74	At 12 Months FU N_2_ = 70	At 24 Months FU N_2_ = 64	Total
Post-Void residual volume	6 (4.13%)	4 (5.63%)	0 (0.00%)	0 (0.00%)	4 (5.63%)	2 (2.70%)	0 (0.00%)	0 (0.00%)	2 (2.70%)
Dyspareunia	6 (4.13%)	0 (0.00%)	0 (0.00%)	0 (0.00%)	0 (0.00%)	1 (1.35%)	4 (5.71%)	1 (1.56%)	6 (8.10%)
Mesh Erosion	4 (2.75%)	0 (0.00%)	0 (0.00%)	0 (0.00%)	0 (0.00%)	0 (0.00%)	1 (1.42%)	3 (4.68%)	4 (5.40%)
Chronic pelvic pain	4 (2.75%)	0 (0.00%)	1 (1.40%)	0 (0.00%)	1 (1.40%)	0 (0.00%)	2 (2.85%)	1 (1.56%)	3 (4.05%)

N = total number of patients; N_1_ = number of patients for Burch group; N_2_ = number of patients for TOT group; FU = Follow-up.

## Data Availability

Data are contained within the article.
